# 

**Published:** 1960-07

**Authors:** 


### MEDICAL JOURNAL OF THE SOUTH-WEST

*(Incorporating the Bristol Medico-Chirurgical Journal)*

Established 1883

VOL. 75 (iii) July 1960 NO. 277

### *Contents*

MOUNTAINS . . . . . *Dr. F. W. Terrell Hughes* 43

MANAGEMENT OF SEPSIS . . . *Herbert K. Bourns* 50

X-RAYS IN EVERYDAY LIFE . . . *J. H. Middlemiss* 55

NEPHROTIC SYNDROME WITH STEATORRHOEA . . . *Clinical Pathological Conference* 64

NOTES AND NEWS . . . . . . . 74

EXAMINATION RESULTS . . . . . . 74

PRIZES . . . . . . . . . 75

APPOINTMENTS . . . . . . . . 75

*PUBLISHED QUARTERLY*

*ANNUAL SUBSCRIPTION* 16/- *POST FREE*

SMITHSONIAN INSTITUTION AUG 25 1960

PUBLISHED UNDER THE AUSPICES OF THE
BRISTOL MEDICO-CHIRURGICAL SOCIETY

### Editorial Committee

Dr. J. E. Cates, Department of Medicine, Bristol Royal Infirmary. *Editor.*
Dr. N. J. Brown, Southmead Hospital, Bristol. *Assistant Editor.*
Dr. A. B. Raper, Department of Pathology, Bristol Royal Infirmary. *Assistant Editor.*
Dr. J. Macrae, Ham Green Hospital.
Dr. R. C. Wofinden, Central Clinic, Bristol.
Mr. A. E. S. Roberts, Medical Library, University of Bristol. *Secretary.*

### *Local Correspondents*

*Bath* . . Mr. A. D. Bateman, 3 The Circus, Bath.
*Exeter* . . Dr. L. N. Jackson, Mount Jocelyn, Crediton, Devon.
*Gloucester* . Dr. R. F. Jarrett, 9 College Green, Gloucester.
*Plymouth* . Dr. C. R. Croft, Lexden, Hartley Av., Plymouth.
*Taunton* . Dr. M. McAlley, 1 The Crescent, Taunton.
*Torquay* . Dr. A. Robinson Thomas, 20 Devon Square, Newton Abbot, Devon.
*Truro* . . Dr. F. D. M. Hocking, St. Andrews, Perranporth, Cornwall.
*Yeovil* . . Mr. J. A. Vere Nicoll, Knapp House, Yeovil, Somerset.

### NOTICE TO CONTRIBUTORS

Original articles are invited, provided they have not been published elsewhere. Notes of forthcoming events, meetings held, degrees conferred, staff appointments, and other local news are welcomed. The editorial committee reserves the right to make literary corrections.

Illustrations should always be supplied ready for reproduction. All re-touchings or other operations required will be charged to the author. Line drawings should be drawn in Indian ink. We regret that we must ask authors to pay for making blocks, if this is necessary.

J. W. ARROWSMITH LTD., WINTERSTOKE ROAD,
BRISTOL.

### *Notes and News*

F.R.C.O.G.: Marjorie O. Bennett.

Professor A. V. Neale has been appointed President of the British Paediatric Association.

Professor A. I. Darling, in June, gave an address to the Congress of European Organisation for Research on Fluorine and Dental Caries Prevention.

Dr. Macdonald Critchley gave the centennial oration during the centenary celebrations in June of the National Hospital, Queen Square.

Dr. R. J. A. Berry, formerly Professor of Anatomy and Dean of the Faculty of Medicine, Melbourne University, has been awarded an Emeritus Professorship by the University Council.

Surgeon Captain J. L. S. Coulter has been elected a F.R.C.S.

### *Examination Results*

*M.D. Cambridge*: Dr. Clifford Evans.
*M.D. Glasgow*: Dr. J. Sluglett.

### UNIVERSITY OF BRISTOL

### *M.D.*

D. H. Bowden
W. K. Metcalf

### *Ph.D.*

Dinah Payne

### *D.P.H.*

Addis, S.

Blancarte, J.

Brzezinski, Z. J.

Ducksbury, Christina F. J.

Mair, Marjorie

McGregor, C. M.

Osman, I. O.

Taylor, R. Y.

### *M.B., Ch.B.*

WITH SECOND CLASS HONOURS

Draisey, T. F.
Ferguson, D. R. (*with Distinction in Surgery*).
Marsh, Margaret E. (*with Distinction in Public Health*).
Morley, Jean L. (*with Distinction in Public Health*).
Morris, Barbara, M.
Neale, G. (*with Distinction in Child Health, and in Public Health*).
Stentiford, N. H.

PASS

Beddard, J. B.

Beesley, Barbara

Bisson, Margaret J.

Bisson, P. G.

Blandford, A. G.

Clarke, R. J.

Cottrill, P. H.

Critchley, Sheila A.

Daniel, A. R.

Dash, J. R.

Dyall, C.

Flaherty, T. H.

Gay, M. J.

Gorman, B. J.

Gurney, P. W. V.

Hackman, B. W.

Hayward, C. J.

Hinchlciffe, A.

Langley, A. G. S.

Malkin, Isobel C.

Miles, Mary P.

Pitman, R. H.

Price, Isabel M.

Rawlins, J. C. D.

Rich, W. J. C. C.

Sebire, J. N.

Taylor, P. H.

Warnecke, D. P.

Whatley, R. J.

White, D. M. D.

Williams, B. E. O.

Winslade, J. R.

### Prizes

*The Edgeworth Prize*: P. Breddy.

*The Edward Fawcett Prize*: Miss G. M. Duffin.

*The Foyle Prize*: D. J. Moffatt.

*The Mackie Pathology Prize*: Mrs. B. M. Morris.

*The Richard Clarke Prize*: G. Neale and Miss J. Morley.

### Appointments

Dr. A. E. Read has been appointed Lecturer in Medicine, University of Bristol.

Dr. T. E. Oppé has been appointed Assistant Director of the Paediatric Unit, St. Mary's Hospital, London.

P. O. Nicholas, M.B. (Bristol), D.C.H., has been appointed Senior Assistant M.O. and School M.O., Castleford and Normanton, Yorkshire.

### *UNITED BRISTOL HOSPITALS*

*February–June*, 1960

| *Name* | *Appointment* | *Formerly* |
|---|---|---|
| READ, A. E., M.D.(LOND.), M.R.C.P.(LOND.). | Assistant Physician (Hon. Contract) in the United Bristol Hospitals during tenure of the post of Lecturer in Medicine in the University of Bristol. | Senior Medical Registrar and Tutor in Medicine, Postgraduate Medical School of London and Hammersmith Hospital. |
| OPPÉ, T. E., M.B., B.S. (LOND.), M.R.C.P., D.C.H. | Assistant Paediatrician (Hon. Contract) in the United Bristol Hospitals during tenure of the post of Lecturer in Child Health in the University of Bristol. | Lecturer in Child Health, University of Bristol. (Hon. Contract, Senior Registrar status in the United Bristol Hospitals.) |
| BERRY, D. C., M.D.S. | Assistant Dental Surgeon (Hon. Contract) in the United Bristol Hospitals during tenure of the post of Lecturer in Prosthetics and Dental Surgery in the University of Bristol. | Lecturer in Prosthetics and Dental Surgery in the University of Bristol. (Hon. Contract, Senior Registrar status in the United Bristol Hospitals.) |
| MOBAREK, A. L., M.B., CH.B.(CAIRO), D.M.R.E. | Registrar of Radiotherapy. | Resident at the Radiology Department, Cook County Hospital, U.S.A. |

